# Supplementary material for: Diminished Immune Response and Elevated Abundance in Gut Microbe Dubosiella in Mouse Models of Chronic Colitis with GBP5 Deficiency
Source: Biomolecules. 2024 Jul 20;14(7):873. doi: 10.3390/biom14070873 (PMC11274912; doi:10.3390/biom14070873)
Supplement: Supplementary file 1 [file biomolecules-14-00873-s001.zip › biomolecules-3116785-supplementary.pdf]

Table S1. Disease activity index (DAI) for assessment of DSS colitis severity[1].

Weight loss (%)	Stool consistency	Rectal bleeding	Score
0	well-formed pellets	hemoccult negative	0
1-5	pasty, semi-formed pellets	hemoccult positive	1
5-10			2
10-15	liquid stools	gross bleeding	3
>15			4

[1] The final score is the sum of individual scores.

Table S2. Histological score for assessment of DSS colitis[2].

Inflammation	Depth of Injury	Crypt Damage	% Involvement	Score
none	none	none		0
slight	mucosal	basal 1/3	0-25	1
moderate	submucosal	basal 2/3	26-50	2
severe	transmural	only surface epithelium intact	51-75	3
		entire crypt and epithelium lost	76-100	4

[2] The final score is calculated with the sum of scores of first three parameters multiplied by the score of "% involvement".

Table S3. Macroscopic scores for assessment of colon damage.

The damage of colon	Score
no damage	0
hyperemia without ulcers	1
hyperemia and wall thickening without ulcers	2
one ulceration site without wall thickening	3
Two or more ulceration sites	4
0.5 cm extent of inflammation or major damage	5
1 cm extent of inflammation or severe damage	6-10

Table S4. Microscopic total damage score for assessment of DSS colitis[3].

Goblet cell depletion	Crypt abscesses	Destruction of mucosal arthitecture	Extent of muscle thicking	Presence and degree of immune cell infiltration	Score
absence	absence				0
presence	presence	normal	normal	normal	1
		moderate	moderate	moderate	2
		extensive	extensive	extensive	3

[3] The final score is the sum of individual scores.

Table S5. The concentration of cytokines in the colon quantitated by Luminex assay (pg/mL).

**Gene**	**WT, NT**	**KO, NT**	**WT, DSS**	**KO, DSS**
**Eotaxin**	404.79 ± 62.30	300.40 ± 58.15	4152.91 ± 2110.61	1338.91 ± 655.42
**G-CSF**	7.45 ± 4.44	1.78 ± 1.88	84.35 ± 49.34	37.28 ± 17.01
**GM-CSF**	29.49 ± 8.62	27.73 ± 3.86	24.83 ± 13.32	24.18 ± 10.12
**IFN-γ**	16.30 ± 1.21	14.79 ± 2.09	20.95 ± 3.03	11.32 ± 1.24
**IL-10**	43.37 ± 5.33	36.29 ± 7.02	63.92 ± 12.70	59.66 ± 15.12
**IL-12p40**	25.17 ± 3.74	20.38 ± 3.78	295.36 ± 84.81	313.84 ± 138.73

**IL-12p70**	29.69 ± 6.99	36.20 ± 11.86	23.30 ± 2.96	18.64 ± 3.29
**IL-13**	68.49 ± 20.35	54.43 ± 15.38	79.37 ± 17.41	75.24 ± 52.01
**IL-17A**	5.88 ± 0.71	5.08 ± 1.13	27.71 ± 17.05	11.92 ± 2.61
**IL-1α**	9.01 ± 1.36	8.47 ± 1.92	25.68 ± 25.66	15.30 ± 7.66
**IL-1β**	13.32 ± 2.01	11.11 ± 1.26	110.76 ± 89.12	41.00 ± 16.99
**IL-2**	50.06 ± 2.68	42.49 ± 4.41	46.82 ± 4.96	45.98 ± 5.84
**IL-3**	3.66 ± 0.77	4.21 ± 1.64	3.72 ± 0.50	3.07 ± 0.57
**IL-4**	4.31 ± 0.91	3.54 ± 1.25	3.89 ± 0.96	3.79 ± 0.91
**IL-5**	4.55 ± 0.64	3.19 ± 1.79	3.49 ± 0.91	3.58 ± 0.96
**IL-6**	11.23 ± 3.05	20.11 ± 4.84	39.06 ± 8.18	19.65 ± 4.86
**IL-9**	37.06 ± 3.15	31.47 ± 6.49	29.66 ± 4.27	29.84 ± 3.43
**KC**	23.12 ± 3.18	19.59 ± 4.03	160.83 ± 48.87	62.90 ± 35.37
**MCP-1**	150.49 ± 23.39	121.22 ± 32.34	860.06 ± 593.08	323.53 ± 94.55
**MIP-1α**	3.33 ± 0.53	2.42 ± 0.61	75.99 ± 33.63	37.01 ± 12.58
**MIP-1β**	68.80 ± 16.01	47.40 ± 17.55	230.00 ± 183.15	175.11 ± 88.42
**RANTES**	82.53 ± 27.92	45.90 ± 9.87	922.37 ± 947.90	542.40 ± 314.87
**TNF-α**	23.51 ± 4.00	22.30 ± 5.86	38.20 ± 5.82	23.82 ± 2.95

Table S6. The concentration of cytokines in the serum quantitated by Luminex assay (pg/mL).

Gene	WT, NT	KO, NT	WT, DSS	KO, DSS
**Eotaxin**	6686.73 ± 2277.48	6843.23 ± 804.63	5984.01 ± 720.83	7441.73 ± 1104.01
**G-CSF**	495.18 ± 434.08	354.73 ± 53.69	2498.91 ± 849.94	2422.69 ± 1959.88
**GM-CSF**	91.97 ± 86.70	93.46 ± 27.19	86.12 ± 43.17	88.00 ± 37.55
**IFN-γ**	15.54 ± 5.63	20.42 ± 5.42	15.87 ± 10.33	22.02 ± 11.73
**IL-10**	117.29 ± 48.44	155.26 ± 26.78	368.87 ± 327.41	187.64 ± 29.57
**IL-12p40**	945.11 ± 337.81	1051.31 ± 74.36	1303.05 ± 288.68	1779.21 ± 338.87
**IL-12p70**	39.93 ± 30.94	74.87 ± 38.12	49.79 ± 49.21	46.14 ± 59.50
**IL-13**	22.43 ± 44.86	34.36 ± 45.24	134.06 ± 111.76	289.28 ± 85.08
**IL-17A**	69.22 ± 39.84	107.52 ± 37.38	82.60 ± 48.07	49.03 ± 43.41
**IL-1α**	19.04 ± 5.90	21.09 ± 10.92	10.26 ± 7.62	25.80 ± 6.20
**IL-1β**	11.50 ± 6.02	23.94 ± 23.81	17.00 ± 6.64	12.60 ± 4.50
**IL-2**	4.91 ± 9.81	0.00	0.00	19.74 ± 28.82
**IL-3**	5.55 ± 4.66	17.30 ± 14.13	7.88 ± 5.00	7.46 ± 3.55
**IL-4**	3.66 ± 2.54	6.37 ± 2.28	4.72 ± 3.81	6.74 ± 3.22
**IL-5**	2.55 ± 2.08	4.93 ± 3.85	124.20 ± 237.01	7.89 ± 6.11
**IL-6**	1.50 ± 2.99	6.62 ± 13.23	20.12 ± 10.05	12.04 ± 7.95
**IL-9**	31.99 ± 20.47	35.29 ± 18.73	21.55 ± 25.53	33.92 ± 24.12
**KC**	106.51 ± 26.05	110.20 ± 9.11	200.82 ± 79.68	210.82 ± 68.47
**MCP-1**	555.66 ± 493.66	633.85 ± 722.28	410.34 ± 238.21	356.31 ± 210.13
**MIP-1α**	4.68 ± 0.52	5.53 ± 4.93	2.42 ± 3.20	3.47 ± 2.21
**MIP-1β**	241.29 ± 120.57	290.48 ± 67.90	222.03 ± 122.36	255.31 ± 48.95
**RANTES**	339.19 ± 35.25	336.66 ± 24.22	307.15 ± 68.50	407.45 ± 94.34
**TNF-α**	45.54 ± 18.43	149.92 ± 150.96	264.70 ± 412.07	48.30 ± 45.45
